# The protocol of a randomized controlled trial for playgroup mothers: Reminder on Food, Relaxation, Exercise, and Support for Health (REFRESH) Program

**DOI:** 10.1186/1471-2458-11-648

**Published:** 2011-08-16

**Authors:** Sarojini MDR Monteiro, Jonine Jancey, Peter Howat, Sharyn Burns, Carlie Jones, Satvinder S Dhaliwal, Alexandra McManus, Andrew P Hills, Annie S Anderson

**Affiliations:** 1 Western Australian Centre for Health Promotion Research, School of Public Health, Curtin University, Western Australia, Australia; 2 Centre for Behavioural Research in Cancer Control, Curtin University, Western Australia, Australia; 3 Curtin Health Innovation Research Institute, Curtin University, Western Australia, Australia; 4 Griffith University and Mater Medical Research Institute, Queensland, Australia; 5 Centre for Public Health Nutrition Research, University of Dundee, Dundee, UK

## Abstract

**Background:**

Mother's physical activity levels are relatively low, while their energy consumption is generally high resulting in 58% of Australian women over the age of 18 years being overweight or obese. This study aims to confirm if a low-cost, accessible playgroup based intervention program can improve the dietary and physical activity behaviours of mothers with young children.

**Methods/Design:**

The current study is a randomized controlled trial lifestyle (nutrition and physical activity) intervention for mothers with children aged between 0 to 5 years attending playgroups in Perth, Western Australia. Nine-hundred participants will be recruited and randomly assigned to the intervention (n = 450) and control (n = 450) groups. The study is based on the Social Cognitive Theory (SCT) and the Transtheoretical Model (TTM), and the Precede-Proceed Framework incorporating goal setting, motivational interviewing, social support and self-efficacy. The six month intervention will include multiple strategies and resources to ensure the engagement and retention of participants. The main strategy is home based and will include a specially designed booklet with dietary and physical activity information, a muscle strength and flexibility exercise chart, a nutrition label reading shopping list and menu planner. The home based strategy will be supported by face-to-face dietary and physical activity workshops in the playgroup setting, posted and emailed bi-monthly newsletters, and monthly Short Message Service (SMS) reminders via mobile phones. Participants in the control group receive no intervention materials. Outcome measures will be assessed using data that will be collected at baseline, six months and 12 months from participants in the control and intervention groups.

**Discussion:**

This trial will add to the evidence base on the recruitment, retention and the impact of community based dietary and physical activity interventions for mothers with young children.

**Trial Registration:**

Australian and New Zealand Clinical Trials Registry ACTRN12609000735257

## Background

Overweight and obesity are important public health concerns. The percentage of Australian women of childbearing age that are overweight or obese has significantly increased over the past decade. In 2007, 44% of Australian women aged between 25 and 34 years were overweight or obese compared to only 26% in 1995 [1].

Childbearing aged women are an important target group for dietary and physical activity interventions as they are at an increased risk of long-term overweight and obesity [2]. Women's increased risk of overweight and obesity after their first and subsequent pregnancies is associated with overweight or obesity prior to pregnancy [3,4], gestational weight gain above the recommended guidelines [5,6], failure to lose gestational weight in an appreciable timeframe or excessive postpartum weight retention [7] and interpregnancy weight gain [8].

Overweight and obese childbearing aged women appear to have a disproportionate risk of maternal, intrapartum, peripartum, neonatal, and postpartum complications [9,10]. If this weight gain continues after childbearing, women will be at increased risk of obesity related chronic conditions such as type II diabetes, high blood pressure, dyslipidaemia, cardiovascular disease and the risk of several major cancers [10]. In addition, maternal obesity may have deleterious effects on the neonate such as macrosomia, increased risk of a range of structural anomalies and of still birth [11].

Research indicates that the mechanisms for interpregnancy and 12 months postpartum weight gain can be due to a range of factors such as lack of nutrition knowledge [12], poor dietary habits and physical inactivity [13,14]. For example, research shows that 96% of females aged 25-34 and 94% of females aged 35-44 consume inadequate fruit or vegetables when compared to the Australian dietary guidelines [1]. Furthermore, despite the known health benefits of physical activity, 30% of women aged between 24 and 34 do not do any exercise, while 44% participate in low intensity activity [1].

The barriers to mothers adopting the recommended physical activity behaviours include lack of social support, lack of time, lack of energy and motivation, procrastination, lack of self-efficacy and childcare and financial constraints [15,16]. The influences on eating habits include convenience, cost, lifestyle preferences, confusion around food messages, nutrition knowledge and environmental factors [17]. Furthermore, common postpartum physical symptoms such as fatigue, headaches, nausea, backache and urinary or bowel problems can inhibit mothers following a healthy diet and physical activity plan [18].

Mothers are an important group within the family unit as they are generally the primary caregiver and help to shape the attitudes and behaviour of their children with respect to food and physical activity. Overweight and obese children are twice as likely to become overweight and obese adults when compared to normal weight children [19]. Mothers can prevent children from becoming overweight and obese as they play a major role in determining the family mealtime environment, and managing the amount and type of food available [20]. Thus, efforts to interrupt this cycle of obesity by targeting interventions at mothers are vital from both a public health perspective. Dietary and physical activity interventions could provide benefits to the mother, her future pregnancies and subsequent generations from becoming overweight and obese [21].

Currently, there are few studies that have reported the effectiveness of behavioural interventions designed to improve physical activity and dietary behaviours [22-26] in mothers with young children. These studies have included small samples and have incorporated limited evaluation measures [23-25], even though the evidence suggests that after childbirth mothers are ready to change behaviours associated with overweight and obesity [27,28].

This paper describes the protocol of a randomized controlled trial to improve the physical activity and nutrition behaviours of mothers with young children (between 0 and 5 years of age) attending playgroups.

## Methods/Design

### Study design

The study is a community based 12 month randomized controlled trial. The study is designed according to the recommendations of the CONSORT statement for randomized trials of nonpharmacologic treatment [29].

**Figure 1:**
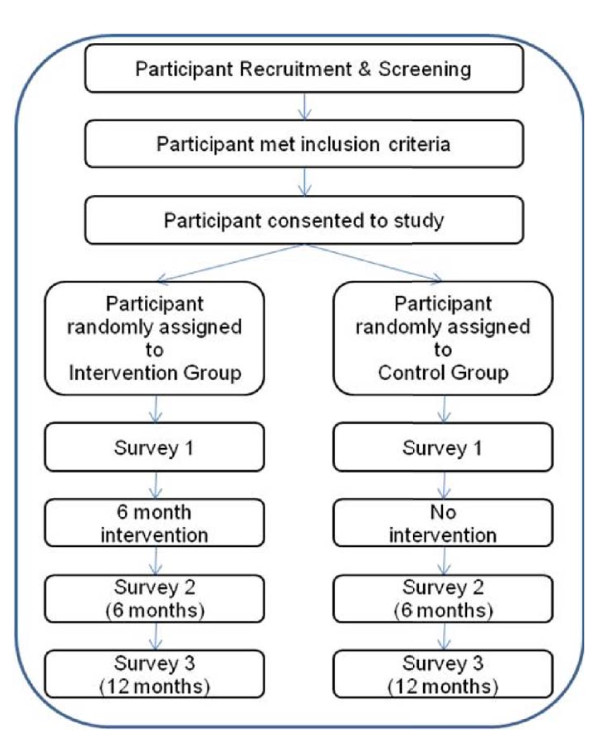
**REFRESH Study Design**.

The REFRESH study will be conducted over three years (Figure 1). The first year will include formative research, development of the evaluation framework and the intervention. In the second year, participants will be recruited, the intervention will be implemented and data will be collected from participants. The final year will include data collection, data analysis and review of the intervention.

### Study aim

The REFRESH study aims to evaluate the effect of a six month physical activity and nutrition randomized controlled trial for mothers with young children attending playgroups in Perth, Western Australia (WA).

The REFRESH program will focus on behaviour change to meet the Australian physical activity guidelines, by encouraging increased in levels of vigorous, and moderate physical activity, the number of steps taken each day and muscle strength exercises [30,31]. The REFRESH program will also aim to encourage behaviour change to meet the Australian dietary guidelines (improve nutritional intake by increasing fruit, vegetable and fibre intake and decreasing fat and added sugar intake) [32].

### Settings

Playgroups in Australia are informal regular community groups that are set up for babies, toddlers and pre-school children (0 to 5 years). The purpose of a playgroup is to encourage play among children to enhance their social, emotional, physical and intellectual development. Parents and carers also find it a valuable resource as they help establish support networks. Playgroups are run by volunteer parents and carers who get together once a week for a couple of hours. They are held at a variety of venues such as libraries, child and maternal health centres, church halls, kindergartens and schools. Playgroups are supported by National and State organisations [33]. The REFRESH project will be conducted in collaboration with Playgroup WA Inc. [34], as the playgroup will be used as the setting to recruit mothers and implement the project.

### Recruitment and randomisation process

A stratified random sampling procedure will be adopted to recruit participants from 560 playgroups embedded in 106 suburbs (neighbourhoods) within the Perth metropolitan area. Stratification will be conducted by suburb geographical location and Socio-Economic Indexes For Area (SEIFA) scores. SEIFA scores are values derived from income, education level, employment status and skill level [35]. The suburbs will then be randomly assigned to either the intervention group or the control group using a table of random numbers. Control and intervention group suburbs will be arbitrarily matched for low and medium levels of socio-economic status based on the SEIFA scores. The senior Playgroup WA Inc. staff will make phone calls to all registered playgroup leaders, explain the REFRESH project and obtain permission for project staff to contact the playgroup. Project staff will visit the playgroup to further explain the project, obtain consent and allocate participants to the intervention or control group. Intervention group participants will also complete the Physical Activity Readiness Questionnaire [36] and provide a medical certificate if deemed necessary before commencing the program.

### Inclusion criteria

Study participants will need to be: (a) women aged 18 or over registered with Playgroup WA Inc.; b) have a child between 0 to 5 years; (c) healthy to the extent that participation in a low-stress physical activity program would not place them at risk; (d) not taken part in any research that involves physical activity or nutrition within the last five years; and (e) not on a special diet.

### Sample size determination

In order to detect a 20% difference in physical activity at 80% power and 5% level of significance, sample size of 310 mothers of young children are required at the 6-months post-intervention survey in each of the intervention and control groups. A small effect size (0.2) [37] is assumed for studies on behavioural effects due to the influence of extraneous variables and the subtleties of human performance. Allowing for an attrition rate of 30%, 900 mothers of young children will be recruited into the study. Sample size calculations were determined using Power Analysis and Sample Size software [38].

### Data collection

Process data will be collected during the implementation of the intervention. The playgroup is a novel setting for the recruitment and delivery of health promotion interventions for mothers with young children. Therefore, the process evaluation will be a key component of the program's evaluation. This will be conducted with both the participants and the project staff, providing two perspectives on the program delivery and content.

Outcome data will be collected at baseline, six months and 12 months. At baseline control group participants will be hand delivered a self-completion questionnaire at the playgroup along with a self-addressed envelope and measuring tape to record waist and hip measurements. The intervention group will be provided all of the above and a pedometer to record the number of steps taken each day. At six months the control group participants will be hand delivered a self-completion questionnaire at the playgroup along with a self-addressed envelope. The intervention group will be provided all of the above and a pedometer to record the number of steps taken each day. At 12 months the control and intervention group participants will be posted a self-completion questionnaire with a self-addressed envelope.

### Blinding

It is not possible to blind study project staff to the randomisation process, however, the participants will be blinded as to whether they are in the study or control group. The assessor will be blinded until the comparative data analysis is conducted. Participants will be given codes when recruited and these codes will be used throughout the implementation of the study. The participant codes will be revealed only at the six and 12 month comparative data analysis.

### Statistical analysis

Data collected will be coded and analysed using the Statistical Package for the Social Sciences computer statistical software [39]. Descriptive statistics will first be used to summarise participants' demographic and health characteristics. For the hierarchical data (repeated measurements of individuals) collected over the one-year observational period, multi-level repeated measures analyses and multivariate logistic regression analyses will be used extensively in the statistical analyses.

### Intervention group

To facilitate the development of the intervention and to ensure adherence to its timeline, the implementation of the intervention will be organised into four stages.

#### Stage 1

##### Intervention development

A literature review of nutrition and physical activity community based interventions for mothers with young children, pregnant and postpartum women has been conducted and will be continuously updated. Relevant behaviour change theories reviewed including the Social Cognitive Theory (SCT) [40], Transtheoretical Model (TTM) [41] and motivational interviewing [42] will support the development of a multi-strategy intervention [43]. Previous qualitative data obtained from Perth playgroup mothers will be used to ascertain the barriers and facilitators to healthy eating and being physically active, as well as their preferred intervention strategies [44]. The Precede-Proceed model will be used to organise the behaviour change theories and formative research data into an appropriate nutrition and physical activity behaviour change program [45,46].

#### Stage 2

##### Recruitment of staff

The program will be staffed by Health Science graduates, who will deliver the face-to-face workshop styled information and skill building sessions. Recruitment of the health promotion, nutrition and sport science graduates will be conducted via local universities and relevant professional associations.

#### Stage 3

##### Staff training

Staff will receive intensive training on the application of the Australian dietary and physical activity guidelines [31,32], and behaviour change theories including motivational interviewing. They will receive a comprehensive training manual on the delivery of the face-to-face workshop sessions. The staff will also receive ongoing support via email and phone by an accredited dietician, human movement specialist, health promotion specialist and the project coordinator.

#### Stage 4

##### Delivery of intervention in playgroup settings

The intervention will be delivered over six months. Interventions that aim to address multiple risk factors such as nutrition and physical activity show more positive outcomes when multiple intervention strategies are used to reach the target audience [43,47]. Hence the intervention group participants will receive four strategies: face-to-face workshop information and skill development session; mailed or emailed newsletters; SMS reminders on the main messages of the REFRESH program; and a home-based component.

##### Delivery of face-to-face workshops

The intervention group participants will receive six workshop sessions over six months (one session a month). Each session will be conducted for 30 minutes by project staff during the playgroup session at the playgroup venue. Workshops will focus on enhancing knowledge, attitudes and skills to enable informed decision making about nutrition and physical activity behaviours (Table 1).

**Table 1:** REFRESH Intervention

Session (Week)	Session Details	Participant resources/interactive activities
1 (Week 1)	1. Introduction to Refresh Program 2. Overview of healthy eating and being physically active 3. Focus nutrition: fruits, vegetables and water • Guidelines • Benefits/barriers/overcoming barriers	1. Resources: • Program booklet • Healthy recipe booklet • Session one information summary pamphlet 2. Interactive activity: • Determine participant program needs

2 (Week 5)	1. Focus behaviour change: • Stages of change • Goal setting: long and short term goals 2. Focus physical activity: aerobic • Guidelines • Benefits/barriers/overcoming barriers 3. Focus nutrition: five food groups and 'extra' foods • Guidelines • Benefits/barriers/overcoming barriers	1. Resources: • Pedometer • Family dinner and physical activity planner (fridge magnet) • 'Extra' food record sheet • Session two information summary pamphlet 2. Interactive activity: • Playgroup 10,000 steps per day challenge

3 (Week 9)	1. Focus behaviour change: • Review established short term goals • Set new short term goals 2. Focus physical activity: Muscle strength and flexibility exercises • Guidelines • Benefits/barriers/overcoming barriers	1. Resources: • Muscle strength and flexibility exercise card (fridge magnet) • Physical activity diary • Session three information summary pamphlet 2. Interactive activity: • Muscle strength and flexibility exercises • Integrated exercises "

4 (Week 13)	1. Focus behaviour change: • Review established short term goals • Set new short term goals 2. Focus nutrition: • Healthy eating messages • Menu planning • Food label reading • Making sense of nutritional claims	1. Resources: • Shopping list with healthy shopping tips • Comparing packaged food per 100 g (fridge magnet) • Session four information summary pamphlet 2. Interactive activity: • Reading packaged food labels • Developing a daily menu

5 (Week 17)	1. Focus behaviour change: • Review established short term goals • Set new short term goals • Overcoming relapses 2. Focus nutrition: fats and sugars • Recommended intake • Benefits/barriers/overcoming barriers	1. Resources: • Session five information summary pamphlet 2. Interactive activity: • Modifying recipes • Healthy cooking methods

6 (Week 21)	1. Focus behaviour change: • Review established short term goals • Social support 2. Focus nutrition: Fibre and Glycemic Index • Recommended intake • Benefits/barriers/overcoming barriers	1. Resources: • Session six information summary pamphlet 2. Interactive activity: • Modifying recipes • Healthy cooking methods

##### Delivery of newsletters

The intervention group participants will receive six newsletters via post or email over six months (one newsletter a month, one week after each face-to-face workshop session). The newsletters will be in an informal format and will contain myth dispelling information on nutrition and physical activity.

##### Delivery of SMS reminders

The intervention group participants will receive 18 SMS reminders via mobile phones over six months (reminders to attend the face-to-face workshop sessions and nutrition and physical activity motivating messages).

##### Delivery of home based component

The intervention group participants will receive home based resources at each of the face-to-face workshop sessions to support the content of the REFRESH program. The home based components will include a specially tailored program booklet, pedometer, menu planner fridge magnet, a shopping list with food label reading tips, a muscle strength and flexibility exercise chart fridge magnet, a physical activity diary and an 'extra' food record sheet. The workshops will offer an opportunity for these resources to be explained and for questions to be answered.

### Control group

The control group participants will not receive any intervention materials. Their only contact with the project will include completing the questionnaires at the three data collection periods.

### Process measures

#### Participant process evaluation

The REFRESH booklet will be assessed by the participants in terms of attractiveness, comprehension, personal relevance, believability, and legibility [48]. Workshop and staff feedback sheets will be provided to participants to assess the content and workshop delivery methods in the playgroup setting. Participants will be invited to comment on the REFRESH program's impact on their physical activity and nutrition behaviours and to provide suggestions for improvements to the intervention [49].

#### Staff process evaluation

The staff will provide feedback on the playgroup as a setting for health promotion programs. This evaluation will focus specifically on the playgroup characteristics, and the skills deemed necessary to deliver workshops in this setting. Staff will also provide feedback on working with mothers as a target group within the playgroup setting, what the mothers want to learn about nutrition and physical activity and common myths mothers report. Staff will also maintain a diary of their perceptions related to the delivery of the face-to-face workshop sessions, and responses by participants to the session content and activities.

### Outcome measures

The self-administered questionnaire will be comprised of instruments which have been previously validated and tested for reliability [50-52], and will undergo further reliability testing prior to its use at baseline.

Physical activity will be measured by The International Physical Activity Questionnaire (IPAQ) [53]. This instrument has been accepted as the physical activity measurement tool in many settings and is specifically designed for population-based prevalence studies. Muscle strength exercise assessment will be based on recommendations from the American Heart Association and Australian physical activity guidelines [54]. Physical activity knowledge will be assessed by a modified version of the American Adult's Knowledge of Exercise Questionnaire [55]. Dietary intake will be measured using a modified version of the Fat and Fibre Barometer [56]. The New South Wales Government questionnaire will be used to measure soft drinks, fruit juice and snack consumption [57]. Added sugar consumption will be assessed using the 2005 National Health Interview Survey [58]. Nutrition knowledge will be assessed by a modified version of the General Nutrition Knowledge Questionnaire [59].

Self-efficacy for nutrition and physical activity behaviours will be assessed. Nutrition and physical activity self-efficacy will be assessed using items from previously validated instruments [52]. Validated questions will also confirm participants' stages of change regarding fruit and vegetable consumption [60]. Social support for physical activity will be assessed based on items from the Sallis et al. instrument [51].

General physical and mental health will be measured by The Medical Outcomes Study Short-Form Health Survey (SF-8) [61]. SF-8 is a standard international generic instrument of health status. It comprises two summary scales - the physical component summary (PCS) score and the mental component summary (MCS) score.

Demographic characteristics will include gender, age, educational level, country of birth, marital status, socioeconomic status, financial status and co-morbidities. Anthropometric measures will include self-reported height and weight, waist and hip girth. A recent study has confirmed that self report measures are suitable for such studies when a correction factor is applied [62].

Height, weight, waist and hip girth measurements will be conducted by research staff on a random subsample of 100 participants from the intervention group. Calculations of differences between self reported and research staff measured data will be undertaken to identify a correction factor based on the methodology of Dhaliwal et al. [62].

## Ethics

The project protocol has been approved by the Curtin University Human Research Ethics Committee (approval number HR 186/2008).

## Discussion

The REFRESH project is unique in using playgroups for a lifestyle intervention. The playgroup environment is an innovative setting for health promotion for mothers with young children, as it offers an exciting avenue to reach this target group and support behaviour change. The recruitment of participants through playgroups is beneficial as it will encourage all playgroup members to register for the program, thereby not just recruiting those who are motivated to adopt health enhancing behaviour [63].

The program will provide an opportunity for a variety of strategies to be implemented and evaluated. This evaluation data will be collected from participants in their own communities and not in a research centre, making the program relevant to the community based population and not just a clinical group. The project will provide guidelines for the development, implementation and evaluation of a minimal intervention home-based tailored physical activity and nutrition program. The information gathered will be valuable in helping to identify and address the barriers to participating in physical activity and nutrition programs for this target group. The project has the potential, to reduce chronic disease and enhance mental health for mothers of young children in the playgroup setting.

## Competing interests

The authors declare that they have no competing interests.

## Authors' contributions

SM coordinated the project, led the design of the REFRESH program and drafted the manuscript. JJ, PH, SB, CJ, SD, AM, AH and AA designed the study and revised the manuscript. All authors read and approved the final manuscript.

## Pre-publication history

The pre-publication history for this paper can be accessed here:

http://www.biomedcentral.com/1471-2458/11/648/prepub
